# Impact of Matric Potential and Pore Size Distribution on Growth Dynamics of Filamentous and Non-Filamentous Soil Bacteria

**DOI:** 10.1371/journal.pone.0083661

**Published:** 2013-12-31

**Authors:** Alexandra B. Wolf, Michiel Vos, Wietse de Boer, George A. Kowalchuk

**Affiliations:** 1 Department of Microbial Ecology, Netherlands Institute of Ecology (NIOO-KNAW), Wageningen, the Netherlands; 2 Department of Soil Quality, Wageningen University, Wageningen, the Netherlands; 3 Department of Ecological Science, Free University of Amsterdam, Amsterdam, the Netherlands; 4 Institute of Environmental Biology, Utrecht University, Utrecht, the Netherlands; Auburn University, United States of America

## Abstract

The filamentous growth form is an important strategy for soil microbes to bridge air-filled pores in unsaturated soils. In particular, fungi perform better than bacteria in soils during drought, a property that has been ascribed to the hyphal growth form of fungi. However, it is unknown if, and to what extent, filamentous bacteria may also display similar advantages over non-filamentous bacteria in soils with low hydraulic connectivity. In addition to allowing for microbial interactions and competition across connected micro-sites, water films also facilitate the motility of non-filamentous bacteria. To examine these issues, we constructed and characterized a series of quartz sand microcosms differing in matric potential and pore size distribution and, consequently, in connection of micro-habitats via water films. Our sand microcosms were used to examine the individual and competitive responses of a filamentous bacterium (*Streptomyces atratus*) and a motile rod-shaped bacterium (*Bacillus weihenstephanensis*) to differences in pore sizes and matric potential. The *Bacillus* strain had an initial advantage in all sand microcosms, which could be attributed to its faster growth rate. At later stages of the incubation, *Streptomyces* became dominant in microcosms with low connectivity (coarse pores and dry conditions). These data, combined with information on bacterial motility (expansion potential) across a range of pore-size and moisture conditions, suggest that, like their much larger fungal counterparts, filamentous bacteria also use this growth form to facilitate growth and expansion under conditions of low hydraulic conductivity. The sand microcosm system developed and used in this study allowed for precise manipulation of hydraulic properties and pore size distribution, thereby providing a useful approach for future examinations of how these properties influence the composition, diversity and function of soil-borne microbial communities.

## Introduction

Soils are highly heterogeneous systems, containing a wide range of micro-habitats and environmental gradients [1]. This extreme heterogeneity, at a variety of spatial scales [2], [3], offers a large potential for niche differentiation and may be an important factor in realizing the tremendous diversity of microbial communities in soil [4]. Bacteria are distributed heterogeneously within the soil matrix, and distances between individual cells and micro-colonies are often very large in comparison to the size of bacterial cells. Whether resources can be accessed by a given organism depends on the distance between the microhabitats, and, perhaps more importantly, on the level of connectivity between these microhabitats via water films [4]. Soil connectivity depends on the geometry of the pore network, which impacts the distribution of soil water, as well as the hydration status [5]. As the growth of many bacterial species is dependent on the availability of soluble organic compounds, the distribution of cells and the hydraulic connectivity of soil micro-habitats will also have great implications for competitive interactions.

Bacteria are essentially aquatic organisms, as they rely on water for functioning and require water-filled pores or water films for passive and/or active motility [6]. Given their ability to bridge air-filled gaps via their hyphae, filamentous fungi may have distinct advantages over many bacteria in unsaturated soils [7]. At low matric potential, fungi can explore micro-habitats that appear not to be accessible to most bacteria [8], and this may explain the observations that fungal activity often exceeds bacteria activity under these conditions [9]–[11]. Experimental work with *Rhizoctonia solani* confronted with different ratios of air- and water-filled pore volumes in sand provides evidence that having a large fraction of air-filled pores stimulates fungal spread in soils [12]. Most soil-bacterial cell morphologies (e.g. rods, cocci, spirals, etc.) are not adapted to bridging the air-filled spaces that occur in non-saturated soils. However, although bacteria are unable to cross air-filled pores on their own, it has been shown that some motile bacteria can move along fungal hyphae (so-called fungal highways) [13]. Also, it has been demonstrated that some bacterial strains, have the ability to co-migrate with other bacteria along fungal hyphae [14], [15]. Fungal hyphae may thus promote the distribution of motile bacteria in unsaturated soils.

Filamentous actinomycetes represent an exception within the bacterial domain, providing a morphological bridge between bacteria and filamentous fungi [8], and although they are much smaller than fungi, their filamentous growth form could provide similar advantages for the exploration of unsaturated soils. The natural habitat of most actinomycetes is soil, where they typically comprise 1 to 20% of the culturable community [16]. *Streptomyces* is the most abundant genus and encompasses key players in the decomposition of soil organic matter due to the ability to produce a large array of extracellular enzymes such as chitinases, cellulases and hemicellulases [17]. Streptomycetes are also known for producing a vast array of antibiotics, some of which are valuable in medicine and agriculture [18].

We hypothesized that actinomycetes might possess “fungal-like” characteristics with respect to their exploitation of less well connected soils, thereby being able to out-compete non-filamentous bacteria under low connectivity conditions. To address this hypothesis, we investigated the competitive ability of a filamentous bacterium (*Streptomyces atratus*) versus a non-filamentous Gram-positive bacterium (*Bacillus weihenstephanensis*) across a series of defined environmental conditions varying in pore size distribution, moisture and habitat connectivity. The population sizes of the two strains were subsequently tracked over time. In line with our hypothesis, we predicted that *Streptomyces* would have a competitive advantage under conditions of low connectivity and that *Bacillus* would perform best in more well-connected habitat matrices.

## Materials and Methods

### Bacterial Strains

We used two soil isolates, *Bacillus weihenstephanensis* AW02 (NCBI submission ID 1572885) and *Streptomyces atratus* AW01 (NCBI submission ID 1572838), both isolated from the Park Grass Experiment at Rothamsted Research, plot 3 (nil) in August 2009. These strains were chosen because they both represent Gram positive soil bacteria that co-occur in soil and process alternative growth and soil exploration strategies. Both strains were isolated from the same single soil aggregate, which was dispersed in phosphate buffer (pH 6.5), shaken for 30 min followed by 2×1 min sonication. Diluted soil suspension was plated on soil suspension agar prepared from soil taken outside the experimental plot. The soil suspension agar was prepared by weighing 100 g air-dried soil in 900 mL phosphate buffer (pH 6.5), shaking for 30 min, sonicating (Branson 5210 ultrasonic bath) twice for 1 min and subsequent filter-sterilization through 0.2 µm pore size; per liter suspension, 15 g agar (Merck) was added. The strains were identified by 16S rRNA gene sequencing, and these sequences have been deposited under NCBI accession numbers JX944825 and JX944824 for *B. weihenstephanensis* AW02 and *S. atratus* AW01, respectively. To determine growth curves of both strains in liquid medium, six replicates of each strain were grown in 96 well plates in 10% tryptic soy broth (TSB) (Oxoid), and the OD at 600 nm was measured over a period of 20 h. Measurements were taken every 20 min, and the 96-well plate was shaken for 2 min before each measurement.

### Construction of Microcosms

**Figure 1:**
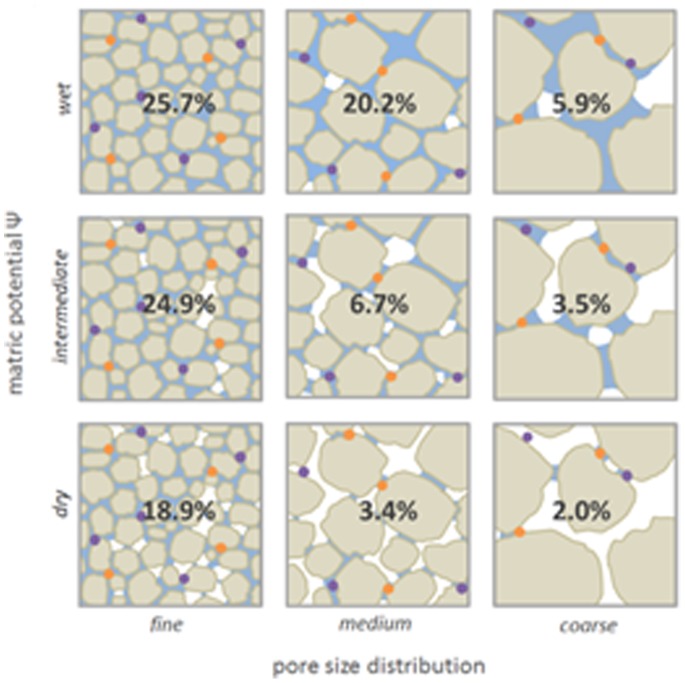
Schematic diagram of the experimental setup. Three matric potentials were combined with three sand particle size fractions with different pore size distributions, giving a total of nine treatments. In each box, the appropriate gravimetric water content of each treatment is indicated. Grain size, water distribution and bacterial cells (orange and purple) are indicated for illustrative purposes and are not based on actual microscopic visualization. Habitat connectivity decreases with decreasing matric potential and increasing pore size. In well-connected soils, (e.g. the treatments “wet” and/or “fine pores”), bacterial species (orange and purple) often inhabit connected microhabitats/pore spaces, thereby allowing for completive interactions. Under less-connected conditions (e.g. low matric potential “dry” and coarse pores), microhabitats are discontinuous, thereby reducing competitive bacterial interactions.

Sand microcosms were constructed using quartz sand particles of different size distributions, obtained by milling (Retsch Mortar Mill RM 200) acid-washed sea sand (Honeywell Specialty Chemicals Seelze GmbH, Seelze, Germany) for 10 min followed by fractionation of the particles into size classes by sieving. Particles of different size fractions were used to create three distinct textures: “fine” (sand particles 53–106 µm), “medium” (106–212 µm), and “coarse” (212–425 µm), thereby creating a range of pore size classes. For the competition experiments, microcosms were established in 100 mL glass vials to which 10 g quartz sand of one of the three particle size fractions was added. For the motility experiments, microcosms were established in glass petri dishes to which 50 g of quartz sand of the different grain size fractions was added. Microcosms were sterilized by autoclaving followed by oven-drying prior to use. To prevent moisture loss, the glass vials were closed with screw cap lids, and the petri dishes were sealed with 2 layers of parafilm after inoculation. The moisture content was set to matric potentials of −10 (“wet”), −20 (“intermediate”) and −50 kPa (“dry”) (see next section). In total, sand microcosms with all nine combinations of three different pore size distributions and three different moisture regimes were established (Fig. 1) with three replicates per treatment and inoculant per time point. Microcosms were weighed after inoculation and at the end of the experiment to confirm that there was no moisture loss.

### Water Retention Curves and Pore Size Distribution

Water is retained in soils largely due to matric forces (adhesive forces between water and solids) in pores and interconnecting pore necks. At saturation, all pores are filled with water, and the matric potential, Ψ, is zero. When the water content decreases, large pores empty first because water is held less tightly adhered to solids in larger capillaries than in smaller ones. The matric potential is more negative when water is adhered more strongly and is thus lower in smaller pores than in larger ones. Consequently, the water content of a soil at a given matric potential depends on the distribution of the pore sizes (diameters). The relationship between matric potential, Ψ, and pore neck diameter, d, is given by the equation Ψ [−kPa]  = 300/d [µm] [19]. Based on this equation, the pore size distribution of a soil can be calculated from the water retention curve (WRC) [20]. The WRC describes the matric potential – water content relationship and can be determined by draining a saturated soil and determining the water content at a given matric potential [21].

Practically, the WRCs of the 3 sand particle size fractions were determined using a pressure plate (15 bar ceramic plate extractor Cat.#1500 and 5 bar ceramic plate extractor Cat.#1600 by Soilmoisture Equipment Corp., Santa Barbara CA,; for pressures of −1580 and −300 kPa) and a ceramic suction table (pF laboratory station, ecoTech Umwelt- Meßsysteme, Germany for pressures −50, −30, −20, −10, −5.3, and −3.7 kPa). Aluminum cylinders with a diameter of approximately 3 cm and a height of 5 cm were completely filled for the suction table and to 1 cm height for the pressure plate. Samples were wetted until saturated, before being drained with the respective apparatus. The decrease in the water content during drainage was calculated from the loss of water volume. All analyses were performed in triplicate. From nine experimentally determined data points (water content at a given matric potential), WRC was determined for each sand particle size fraction according to the model of Van Genuchten [22] in RETC version 6.02. The bulk density of the sand particles in the cylinders was comparable to that used in the microcosms, allowing one to assume that the pore size distribution in the sand microcosms can also be calculated from the water retention curves.

### Motility Measurements

The motility rates of both strains across the range of sand microcosm were measured using a method that was developed to determine expansion of bacteria in soils [23]. Microcosms were established in glass petri dishes, sterilized by autoclaving and oven-drying, and adjusted to the different matric potentials by adding the appropriate volumes of liquid growth medium (10%TSB) and inoculated at the center of the petri dish with 5 µL overnight culture of *B. weihenstephanensis* or *S. atratus*. After 5 h, 23 h and 47 h, bacterial expansion was determined by sampling with a multi-pronged sampler which measures expansion in 4 directions (Fig. S1). The prongs (spaced equally at 2 mm intervals) were first pushed approximately 5 mm into the sand and then onto a TSB agar plate. The soil layer in the petri dish was approximately 1 cm thick; by sampling this way we avoided sampling from the water-film which formed at the sand matrix-petri-dish interface at the bottom of the petri dish. After three days of incubation of the TSB agar plates the transfer of bacteria by prongs was determined and the expansion in the sand microcosm was calculated. All measurements were performed in duplicate, resulting in a total of 8 measurements per treatment and time point (2 replicates, 4 measured directions), of which the mean was used for statistical analysis. A Three-Way ANOVA was performed in SigmaPlot (version 12.3) to test for the effects of pore size distribution (independent variable 1), matric potential (independent variable 2) and time (independent variable 3), on the expansion of both *Bacillus* and *Streptomyces* (radius of extension of each strain = dependent variable).

### Competition Experiments

10% TSB was used as growth medium and microcosms were incubated at 20°C, with all treatments performed in triplicate. Different treatments had different total amounts of nutrients since we have chosen to use similar nutrient concentrations in the liquid phase for all 9 texture-moisture combinations. The alternative to have all combinations with same absolute amount of nutrients would have resulted in strongly different concentrations of nutrients in the liquid phase, which will result in different osmotic pressures. Microcosms were inoculated either with the *Streptomyces* and *Bacillus* strain as pure cultures (10^5^ cells/g soil; based on colony-forming unit (CFU) counts) or with both strains in a 1∶1 ratio with 10^5^ cells/g soil of each strain. The inoculation was performed by adding the appropriate numbers of bacterial cells to the nutrient solution, which was subsequently added to the sand microcosms. Microcosms were tilted, after which the inoculum was pipetted on the bottom of the glass vial. Tilting the microcosms back ensured homogenization of the inoculum. A total of 405 microcosms were constructed, representing 9 treatments, 3 inoculums, 3 replicates, and 5 time points. Sampling was performed destructively after 0, 3, 6, 9, and 12 days by adding phosphate buffer (10 mM, pH 6.5) to a total volume of 10 mL to each microcosm, and cells were suspended by shaking the microcosms for 30 min, followed by sonication (Branson 5210 ultrasonic bath) twice for 1 min. One mL of the resulting supernatant was sampled to make serial dilutions, which were spread on 10% TSB agar plates for the determination of CFU of both strains. As the two strains differ in their colony morphology, they could be easily distinguished on agar plates. A three-way ANOVA was performed in SigmaPlot to test for a possible effect of pore size distribution (independent variable 1), matric potential (independent variable 2) and time (independent variable 3), on the ratio between *Bacillus* and *Streptomyces* (B/S-ratio = dependent variable), which we used as indicator of the competitive strength of the strains. To test the effect of pore size distribution (as generated by the different sand particle sizes), matric potential and the presence of the competitor (*Streptomyces*) on cell densities of *Bacillus* (dependent variable), a three-way ANOVA was performed for each time point (except the starting point T0 = 0 d) using the following parameters: pore size distribution (independent variable 1), matric potential (independent variable 2) and inoculant (independent variable 3). A series of three-way ANOVAs was performed to test the effect of pore size distribution, matric potential and the presence of the competitor (*Bacillus*) on cell densities of the *Streptomyces* (dependent variable).

### Antagonism Assay

As many *Streptomyces* are known to produce antibacterial compounds [24], we also tested for a possible antagonistic effect of *Streptomyces* against *Bacillus*, by performing an agar overlay assay. 2 µL of an overnight culture of *Streptomyces* was spotted on the surface of a 10% TSB agar plate and incubating at 20°C until growth could be observed. The plate was then overlaid with 8 mL 10% TSB soft agar seeded with 50 µL of an overnight culture of *Bacillus*. After incubation, the plates were examined for zones of inhibition.

## Results and Discussion

### Characterization of the Artificial Soil Microcosms: Water Retention Curves

**Figure 2:**
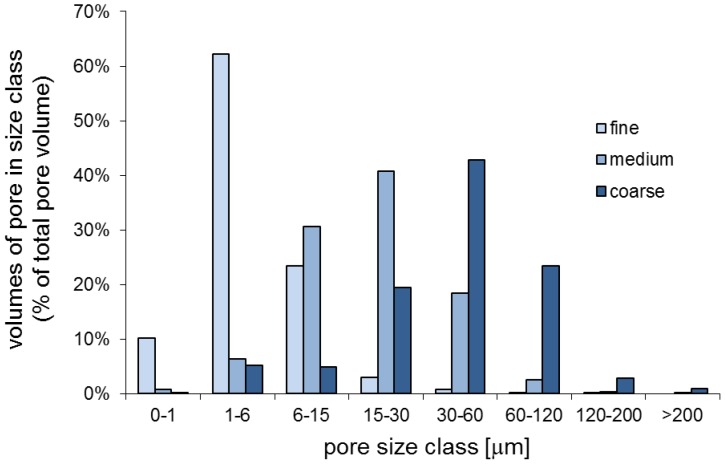
Pore size distribution of the three sand size fractions (fine, medium, coarse). Boxes indicate water-filled pores at different matric potentials, i.e. in the “dry” treatments all pores with a pore-neck diameter < = 6 µm are filled with water, in the “intermediate” treatments < = 15 µm and in the “wet” treatments < = 30 µm, respectively. Pores above these pore-neck diameters are filled with air.

The water retention curves were characterized by the van Genuchten model giving the constants displayed in Table S1 [22]. The pore size distributions of each sand size fraction (as shown in Fig. 2) was calculated from the water retention curves (Fig. S2). Because the relationship between matric potential Ψ and pore neck diameter d is given by the equation Ψ [−kPa]  = 300/d [µm], we could predict which pore sizes would theoretically be filled with water at a specific matric potential (shown in Fig. 2).

### Motility

**Figure 3:**
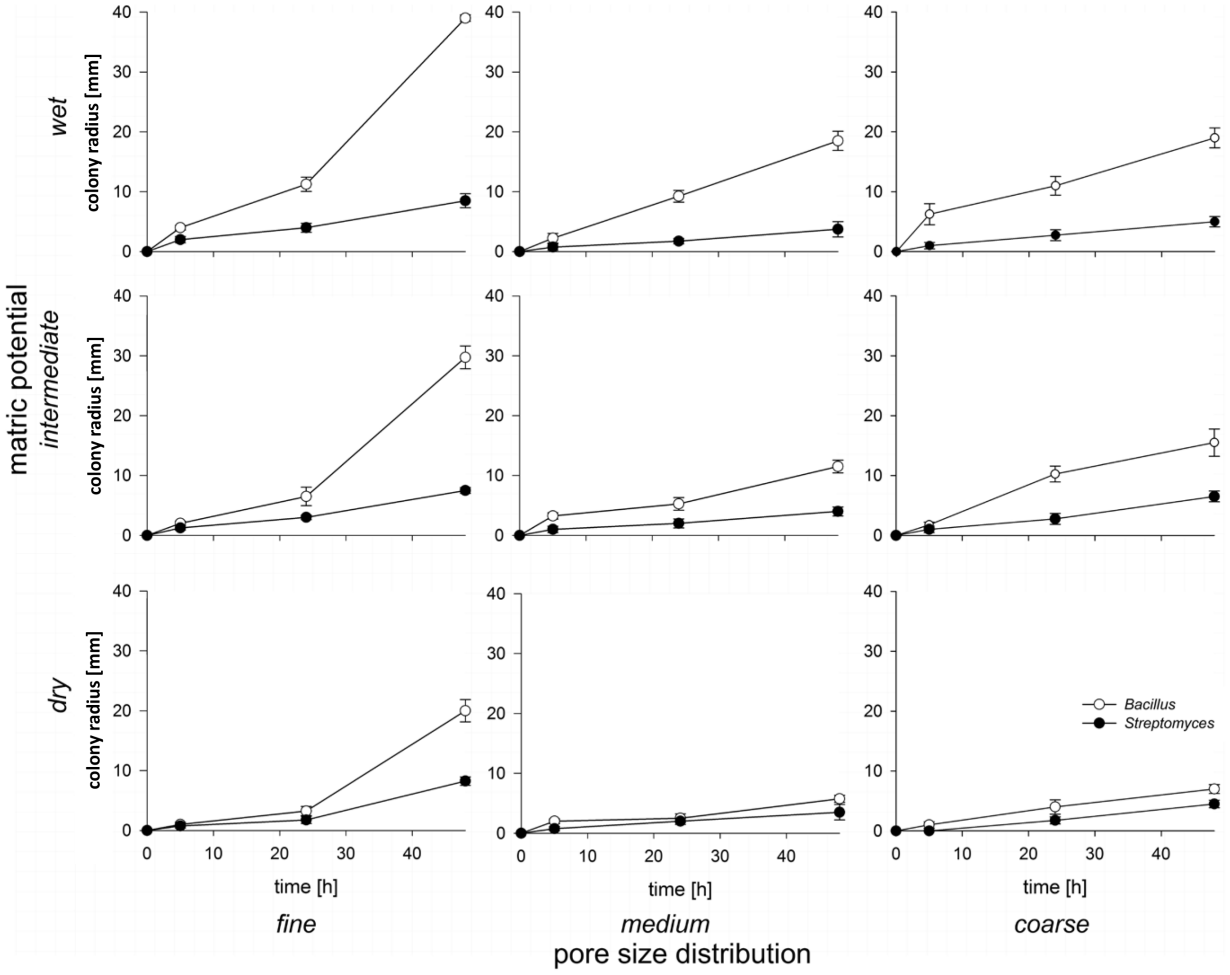
Expansion of *Streptomyces* and *Bacillus* inoculated individually in nine different combinations of pore size distribution and matric potential. Each data point represents the mean of 2 replicate microcosms with 4 measurements per microcosm. Error bars show the standard deviation.

The tests for the expansion ability of the strains in sand microcosms revealed that *Bacillus* displayed greater expansion in sand than *Streptomyces* except for the dry medium and - coarse sand sizes, where expansion of both strains was nearly equal (Fig. 3). This may be attributed to the faster growth rate of *Bacillus* as compared to that of *Streptomyces* as observed for cultures in liquid 10%TSB (Fig. S3). The expansion rate of *Bacillus* was significantly affected by pore size distribution (p<0.001) as well as by matric potential (p<0.001) (Tab. 1). It was fastest in the most connected sand fractions (fine and wet) (Fig. 3). There was also a significant interaction effect of matric potential and pore size distribution on the expansion of *Bacillus* (p<0.001). As hydraulic connectivity is determined by the interplay of hydration status and pore geometry, this demonstrates that habitat connectivity impacts the expansion of *Bacillus*. An explanation for *Bacillus* being more affected by matric potential and pore size is that motility of this strain relies exclusively on the presence of water-filled pores and water films on solid surfaces. The expansion rate of *Streptomyces* was also significantly affected by pore size distribution (p<0.001) and was greatest in the fine sand fraction (Fig. 3). Unlike *Bacillus*, the expansion rate of *Streptomyces* was not significantly affected by matric potential (p = 0.066). Overall, matric potential and pore size distribution had a smaller effect on the expansion of *Streptomyces* than *Bacillus.* We attributed this to the hyphal growth form of *Streptomyces* that allows it to spread through air-filled pores and makes it less dependent on water for its motility.

**Table 1:** Three-way ANOVA of factors affection expansion of bacteria in sand microcosms.

		*Streptomyces*			*Bacillus*		
Source of Variation		DF	F	P	DF	F	P
pore size distribution		2	56.5	**<0.001**	2	68	**<0.001**
matric potential		2	2.8	*0.066*	2	109.4	**<0.001**
time		3	249.5	**<0.001**	2	431.7	**<0.001**
PSD × MP		4	0.8	0.558	4	2	*0.095*
PSD × T		6	17.3	**<0.001**	4	69.4	**<0.001**
MP × T		6	0.8	0.55	4	19.2	**<0.001**
PDS × MP × T		12	0.6	0.883	8	2	*0.051*
Residual		252			189		
Total		287			215		

P values <0.05 (in bold) were regarded as statistically significant and p values <0.1 (in italic) display a non-significant trend towards significance.

### Population Dynamics

**Figure 4:**
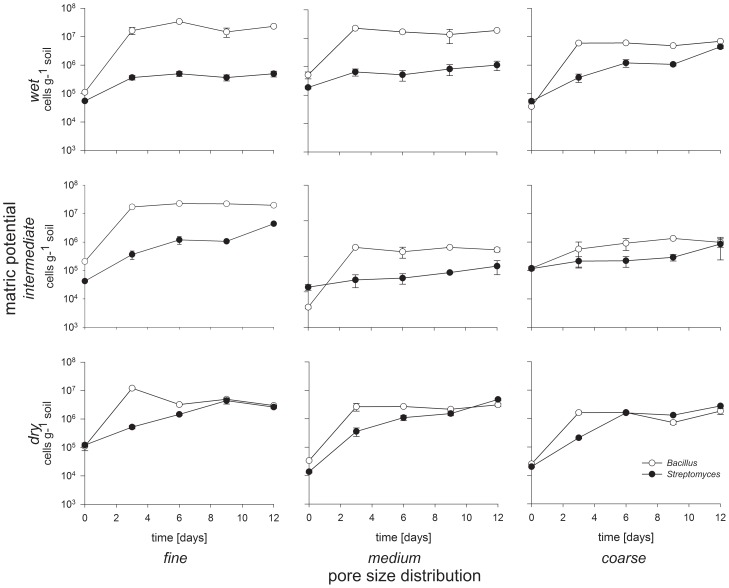
Population dynamics of *Streptomyces* and *Bacillus* competing in nine different combinations of pore size distribution and matric potential. The x-axis is time in days and the y-axis is cell number (g drywt soil) on a log-scale. Each data point is the mean of three microcosm replicates. Error bars show the standard deviation.

In the mixed inoculated microcosms, the rod-shaped *Bacillus* exhibited greater growth than the filamentous *Streptomyces* during the first three days of incubation in all treatments (Fig. 4). This result is in accordance with the faster growth of this *Bacillus* strain in liquid cultures (Fig. 3S). In less-connected conditions (medium and coarse sand combined with dry and intermediate matric potential), *Streptomyces* caught up and ultimately reached higher cell numbers than *Bacillus* (Fig. 4) after 12 days of microcosm incubation. In wetter treatments (see Fig. 1), the numbers of *Bacillus* significantly exceeded the numbers of *Streptomyces* during the whole incubation period: *Bacillus* outnumbered *Streptomyces* by 52-, 22- and 1.6-times in the wet-fine, wet-medium and wet-coarse treatments, respectively, at day 12. In less-connected soils (coarse and dry), *Streptomyces* cells outnumbered *Bacillus* cells at the end of the experiment, e.g. the medium-dry and coarse-intermediate soils had approximately 1.7 and 2.2 times more *Streptomyces* cells than *Bacillus* cells at day 12, respectively.

We found that the population dynamics of the filamentous *Streptomyces* and the rod-shaped *Bacillus* strains were influenced by pore size distribution (p<0.001) and matric potential (p<0.001), as well as the interaction between pore size distribution and matric potential (p = 0.005) (Tab. 2). *Bacillus* cell numbers were not affected by the presence of the *Streptomyces* strain at 3 out of 4 time points, whereas *Streptomyces* was affected by the presence of *Bacillus* at all time points (p<0.001 for all time points; Tab. 3). In line with our hypothesis, the filamentous *Streptomyces* performed best when connectivity was low (coarse pores and dry conditions). Due to its faster growth rate, we anticipated that *Bacillus* would be more successful than *Streptomyces* in the early stages of the experiment. Such a pattern was indeed observed, with *Bacillus* cells outnumbering *Streptomyces* cells after three days of incubation in all treatments (Fig. 4). As the experiment progressed, the *Streptomyces* strain outcompeted *Bacillus* in poorly-connected soils, probably because of the ability of *Streptomyces* to exploit new microhabitats which may still contain nutrients that are inaccessible to the *Bacillus* strain. These results indicate that the hyphal growth may provide a benefit in less-connected matrices by giving the organism access to nutrient patches that cannot be reached by non-hyphal organisms.

**Table 2:** Three-way ANOVA of factors affecting *Bacillus/Streptomyces* (B/S) ratios.

Source of Variation	DF	F	P
pore size distribution (PSD)	2	12.0	**<0.001**
matric potential (MP)	2	18.8	**<0.001**
time (T)	3	2.7	*0.053*
PSD × MP	4	4.1	**0.005**
PSD × T	6	0.6	0.752
MP × T	6	1.1	0.367
PSD × MP × T	12	0.9	0.573
Residual	72		
Total	107		

The ratio between cell densities of *Bacillus* and *Streptomyces* (B/S-ratio) was used as a measure of competitive strength. P values <0.05 (in bold) were regarded as statistically significant and p values <0.1 (in italic) display a non-significant trend towards significance.

**Table 3:** Three-way ANOVAs of factors affecting population densities of *Bacillus* and *Streptomyces* strains after the given periods of incubation.

		*Bacillus*								*Streptomyces*							
Source of Variation/time [d]		3		6		9		12		3		6		9		12	
	DF	F	P	F	P	F	P	F	P	F	P	F	P	F	P	F	P
pore size distribution	2	63.4	<0.001	203.5	**<0.001**	51.1	**<0.001**	315.4	**<0.001**	17.0	**<0.001**	39.9	**<0.001**	17.2	**<0.001**	47.7	**<0.001**
matric potential	2	19.2	<0.001	152.9	**<0.001**	26.3	**<0.001**	346.7	**<0.001**	37.9	**<0.001**	94.8	**<0.001**	8.8	**<0.001**	37.6	**<0.001**
Inoculant (I)	1	1.6	0.220	0.3	0.617	4.5	**0.042**	0.2	0.667	76.6	**<0.001**	314.1	**<0.001**	60.7	**<0.001**	162.1	**<0.001**
PSD × MP	4	13.5	<0.001	43.7	**<0.001**	29.5	**<0.001**	82.5	**<0.001**	19.0	**<0.001**	72.3	**<0.001**	3.5	**0.017**	15.2	**<0.001**
PSD × I	2	1.2	0.300	1.2	0.303	1.3	0.279	19.9	**<0.001**	17.2	**<0.001**	38.4	**<0.001**	14.9	**<0.001**	46.8	**<0.001**
MP × I	2	10.5	<0.001	0.9	0.420	3.0	*0.063*	8.5	**<0.001**	32.7	**<0.001**	87.0	**<0.001**	9.0	**<0.001**	30.8	**<0.001**
PSD × MP × I	4	3.4	0.018	1.9	0.136	4.6	**0.004**	12.8	**<0.001**	16.0	**<0.001**	73.7	**<0.001**	4.1	**0.007**	14.9	**<0.001**
Residual	36																
Total	53																

P values <0.05 (in bold) were regarded as statistically significant and p values <0.1 (in italic) display a non-significant trend towards significance.

The treatments in which *Bacillus* had the greatest competitive advantage coincided with the treatments where this strain also had the greatest advantage in motility (Fig. 3 and 4, Tab. 1, 2, and 3). Treatments in which *Streptomyces* could catch up with or outnumber *Bacillus* were those where the differences in expansion rate between *Bacillus* and *Streptomyces* were smallest. This suggests that motility may have been a particularly important factor in determining the outcome of the competition in the sand microcosms.

Increased motility in well-connected soils also may enable *Bacillus* to more readily colonize new habitats. Remus-Emsermann and colleagues [25] found that the level of pre-colonization of leaf surfaces affected the establishment of a secondary colonizer. Similarly, in the well-connected soil microcosms, microsites are likely to be pre-colonized by the faster-growing and more motile *Bacillus* strain, which may then hamper subsequent colonization by the *Streptomyces* strain.

Overall, the patterns observed in our competition experiments could generally be explained by differences in growth rate, motility and growth form. Although we attributed the relative success of *Streptomyces* in the least connected artificial soil microcosms to its ability to produce hyphae, its ability to produce toxin may also have played a role in its interaction with *Bacillus*. The antagonism assay indicated the production of an inhibiting compound by *Streptomyces*, as zones of inhibition around colonies of *Streptomyces* were observed in our *Bacillus* soft-agar overlay experiment (Fig. S4). Although toxin production was indicated in our soft-agar overlay assay, the growth dynamics observed in our study did not seem to indicate any effects of toxin production under the conditions used. Tracking toxin levels and examining the impacts of soil connectivity on toxin-mediated antagonistic interactions remain interesting issues for future research in bacterial competition.

The hyphal growth form of bacteria has been recognized to be superior over non-filamentous growth forms for the degradation of insoluble polymers such as cellulose and chitin in soils, as hyphae can grow along polymer chains and penetrate into these structures [26]. However, our results indicate another advantage of the hyphal growth of actinomycetes, namely that it enables these organisms span air-filled pores to access nutrients that cannot be accessed by non-filamentous bacteria. This may allow for the co-existence of filamentous and non-filamentous bacteria in soil. Hence, both specialization (e.g. polymer degradation) and habitat exploitation abilities of actinomycetes may contribute to the maintenance of microbial diversity.

Despite the potential importance of soil characteristics that impact habitat connectivity on interactions between (individual) microbes, and thereby ultimately on soil biodiversity, relatively few studies have investigated how soil structure and connectivity affect competition between soil microorganisms. Treves et al. [27] introduced two bacterial species, *Ralstonia eutropha* and *Sphingomonas sp*., competing for a single resource into sand microcosms with different matric potentials and found that both strains could co-exist under dry conditions, but not under wet conditions. The authors concluded that spatial isolation created by low moisture content could contribute to the structuring of soil microbial communities. Similarly, Carson et al. [28] provided evidence that low pore connectivity caused by low water potential could increase the richness and diversity of a complex bacterial community in soil. The observation that soil pore size can impact community composition was also made by Ruamps et al. [29] who used ^13^C-labelled fructose and PLFA to track differential responses related to pore size classes. Whereas these studies focused separately on either the effects of moisture content or pore size, we developed and characterized an artificial sand microcosm system that allows for the independent manipulation of both moisture and pore size distribution, thereby allowing us to address the individual impact of these factors as well their interaction. Such systems should prove useful in helping to disentangle the impacts of various microbial interactions and soil parameters on shaping soil-borne microbial diversity. In contrast to true soils, this system has the advantage that soil structure parameters can be precisely defined and reproduced. The experimental conditions used in our microcosm experiments provided a wide and realistic range of soil pore sizes [30] and matric potentials [31]. Although we used this system specifically to investigate interactions between filamentous and non-filamentous bacteria, it holds the potential to facilitate the examination of other organismal interactions in soil, such as chemical signaling and quorum sensing, grazing and resource competition.

## Supporting Information

Figure S1 **Schematic design for measurements of bacterial motility.** Microcosms containing sand with different pore size distributions and matric potentials were established in glass petri dishes. The microcosms were inoculated in the middle with an overnight culture of either *Streptomyces* or *Bacillus* ( = inoculation point). A multi-pronged sampling device was used at 24 and 48 h to measure the bacterial expansion in four directions by transferring bacterial cells with the prongs from defined distances ( = sampling points) onto agar plates where colony formation was observed.
(DOCX)

Figure S2 **Water retention curves of the 3 sand fractions used in the experiments, showing the water-filled pore space at each matric potential.**
(DOCX)

Figure S3 **Growth curves of** ***Bacillus weihenstephanensis*** **and** ***Streptomyces atratus*** **in 10% tryptic soy broth (n = 6).** Error bars represent the standard error of the mean.
(DOCX)

Figure S4 **Antagonism assay of** ***Streptomyces*** **colonies overlaid with** ***Bacillus*** **in soft-agar.** Zones of inhibition around the colonies indicate the production of an inhibiting compound by *Streptomyces*.
(DOCX)

Table S1 **Constants of water retention curves according to the model of van Genuchten as expressed by the equation S_e_ = [1+(α h)n]^–m^ with S_e_ = (θ_h_ – θ_r_)/(θ_s_ – θ_r_) and m = 1−(1/n). θ_h_ is the soil water content (cm^3^ cm^−3^) at the suction h (cm), θ_r_ and θ_s_ are the residual and saturated soil water contents (cm^3^ cm^−3^).** S_e_ is the effective saturation; the parameters α, m, and n are empirical and determined by a best-fit procedure; α is a parameter related to the inverse of the air entry suction (cm^−1^), n is a dimensionless curve shape parameter and s is the slope of θ_h_ [32].
(DOCX)
