# Supplementary figures and images for: Impact of Matric Potential and Pore Size Distribution on Growth Dynamics of Filamentous and Non-Filamentous Soil Bacteria
Source: PLoS One. 2013 Dec 31;8(12):e83661. doi: 10.1371/journal.pone.0083661 (PMC3877067; doi:10.1371/journal.pone.0083661)

Figure S1 – Wolf et al


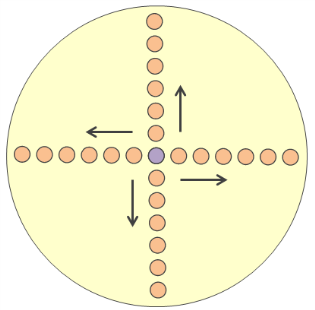


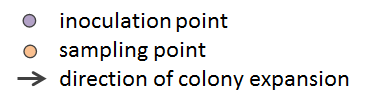

Supplement: Figure S1 — Schematic design for measurements of bacterial motility. Microcosms containing sand with different pore size distributions and matric potentials were established in glass petri dishes. The microcosms were inoculated in the middle with an overnight culture of either Streptomyces or Bacillus ( = inoculation point). A multi-pronged sampling device was used at 24 and 48 h to measure the bacterial expansion in four directions by transferring bacterial cells with the prongs from defined distances ( = sampling points) onto agar plates where colony formation was observed. (DOCX) [file pone.0083661.s001.docx]

Figure S2 – Wolf et al


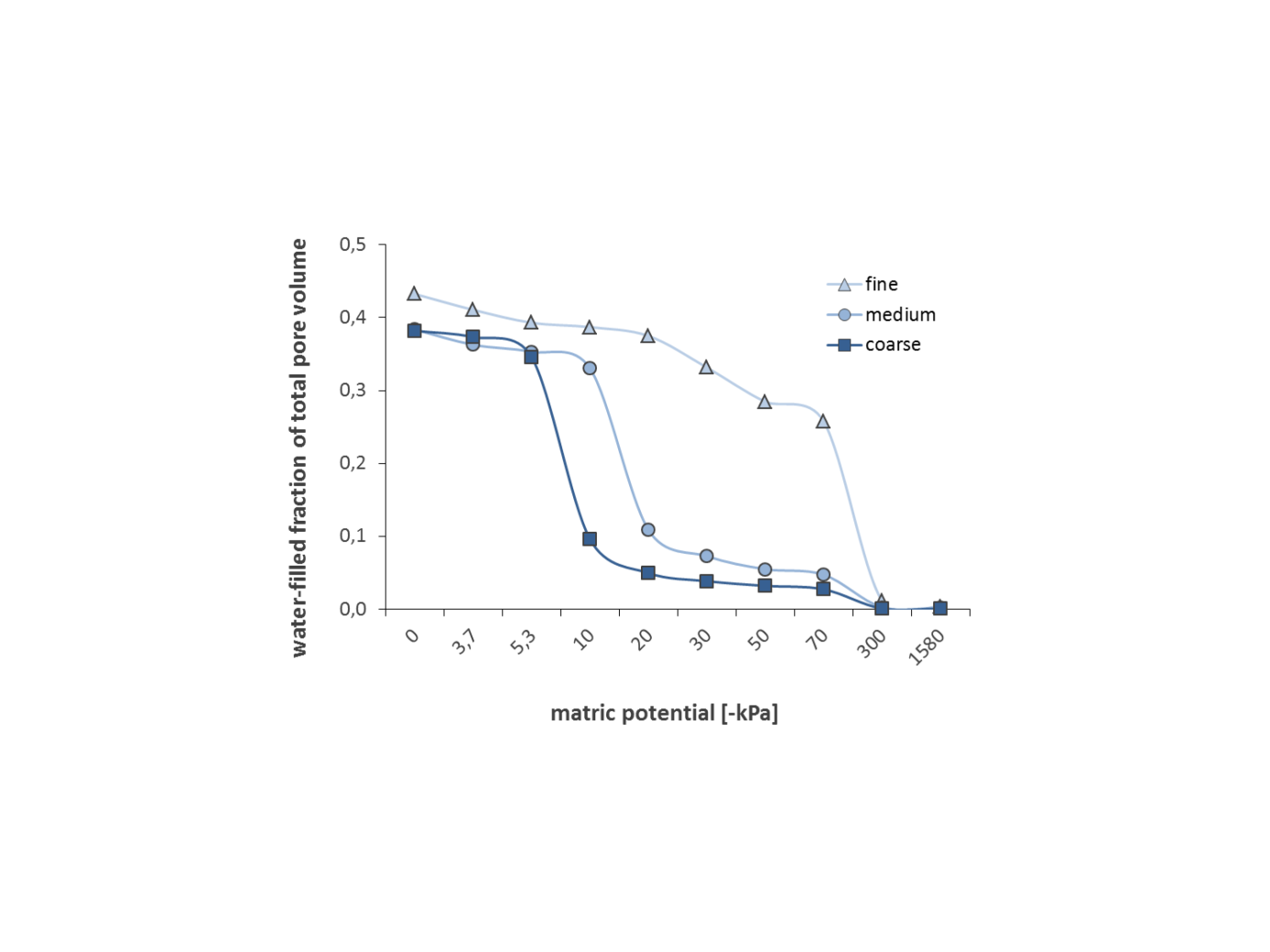

Supplement: Figure S2 — Water retention curves of the 3 sand fractions used in the experiments, showing the water-filled pore space at each matric potential. (DOCX) [file pone.0083661.s002.docx]

Figure S3 – Wolf et al.


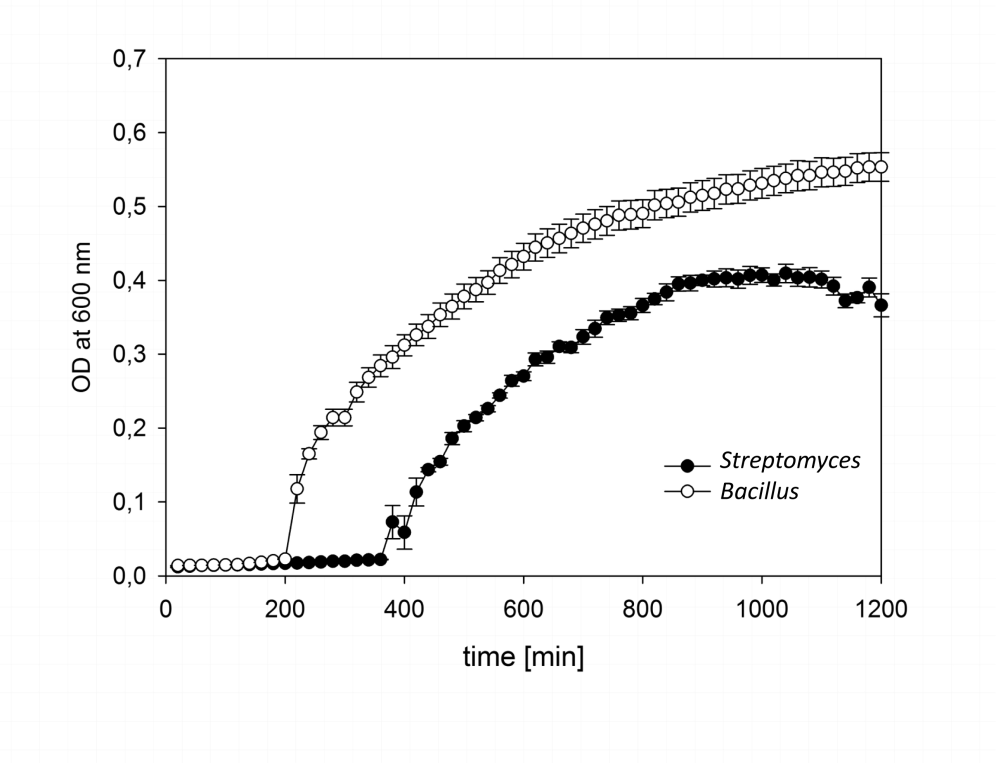

Supplement: Figure S3 — Growth curves of Bacillus weihenstephanensis and Streptomyces atratus in 10% tryptic soy broth (n = 6). Error bars represent the standard error of the mean. (DOCX) [file pone.0083661.s003.docx]

Figure S4 – Wolf et al


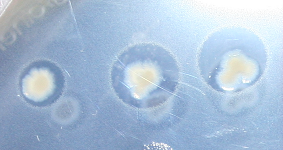

Supplement: Figure S4 — Antagonism assay of Streptomyces colonies overlaid with Bacillus in soft-agar. Zones of inhibition around the colonies indicate the production of an inhibiting compound by Streptomyces. (DOCX) [file pone.0083661.s004.docx]
