# Supplementary material for: Impact of Matric Potential and Pore Size Distribution on Growth Dynamics of Filamentous and Non-Filamentous Soil Bacteria
Source: PLoS One. 2013 Dec 31;8(12):e83661. doi: 10.1371/journal.pone.0083661 (PMC3877067; doi:10.1371/journal.pone.0083661)
Supplement: Table S1 — Constants of water retention curves according to the model of van Genuchten as expressed by the equation Se = [1+(α h)n]–m with Se = (θh – θr)/(θs – θr) and m = 1−(1/n). θh is the soil water content (cm3 cm−3) at the suction h (cm), θr and θs are the residual and saturated soil water contents (cm3 cm−3). Se is the effective saturation; the parameters α, m, and n are empirical and determined by a best-fit procedure; α is a parameter related to the inverse of the air entry suction (cm−1), n is a dimensionless curve shape parameter and s is the slope of θh [32]. (DOCX) [file pone.0083661.s005.docx]

Table S1 – Wolf et al.

| soil type | S | α | n | total porosity [%] |
| --- | --- | --- | --- | --- |
| fine | 0.401 | 0.002 | 2.378 | 40.11 |
| medium | 0.379 | 0.008 | 2.964 | 37.88 |
| coarse | 0.375 | 0.017 | 3.252 | 37.49 |
